# Optimizing noise control in flexible shells with bridging membrane discs variations

**DOI:** 10.1371/journal.pone.0328301

**Published:** 2025-09-05

**Authors:** Hani Alahmadi, Muhammad Afzal, Naif Alkuhayli

**Affiliations:** 1 Department of Mathematics, College of Science, Jouf University, Sakaka, Saudi Arabia; 2 Department of Mathematics, Capital University of Science and Technology, Islamabad, Pakistan; 3 Department of Mathematics and Natural Sciences, Center for Applied Mathematics and Bioinformatics, Gulf University for Science and Technology, Hawally, Kuwait; COMSATS University Islamabad, PAKISTAN

## Abstract

This study explores the acoustic behavior of flexible cylindrical shells incorporating membrane discs at structural interfaces, focusing on their influence on wave propagation characteristics. The dynamics of the embedded membrane discs are modeled at the junctions between different shell segments, and the resulting boundary value problem is addressed using a combination of the Mode-Matching (MM) and Galerkin methods. The governing equations comprise the Helmholtz equation in the fluid domain and the Donnell–Mushtari shell equations in the elastic guiding regions. To ensure the accuracy and convergence of the semi-analytical solution, generalized orthogonality conditions are employed. A truncated modal expansion is used to reconstruct the matching conditions and enforce physical conservation laws at the interfaces. Numerical simulations are conducted to examine the effects of geometric parameters—such as the radii of adjacent shell segments, the size of the membrane discs, and the excitation frequency—providing valuable insights for the design and optimization of waveguide-based acoustic attenuation systems.

## 1 Introduction

Understanding the distribution and dispersion of energy in physical systems is fundamental to a wide array of industrial and mechanical applications. Wave analysis plays a central role in explaining how energy propagates through diverse environments, such as water surfaces, seismic zones, optical fibers, building structures, and locomotive systems. It is also essential in the design and operational efficiency of heating, ventilation, and air conditioning (HVAC) systems. Noise pollution, whether indoors or outdoors, poses a persistent problem in modern settings. Common sources include vehicle exhausts, aircraft engines, power plants, and HVAC installations. To mitigate this, silencers—typically featuring perforated sections—are integrated into exhaust systems to allow acoustic energy to dissipate into surrounding chambers. Although cylindrical waveguides are frequently used, rectangular configurations are also prevalent, thereby prompting extensive research into the acoustic behavior of cylindrical shells. Waveguide systems serve a critical function in air distribution within HVAC frameworks. Nevertheless, fan-induced airflow can produce unwanted noise that propagates through the ductwork. Additionally, external noise may enter and spread within the waveguide system. This necessitates detailed studies on vibration and acoustic propagation in circular cylindrical shells to improve noise control. Of particular interest is how structural irregularities—such as membrane discs and step discontinuities—affect wave transmission. Numerous studies have focused on wave propagation in rigid cylindrical structures. For instance, Miles [1] analyzed acoustic scattering in rigid ducts, emphasizing the role of evanescent modes caused by geometric transitions. Levine and Schwinger [2] derived explicit solutions for reflection coefficients in semi-infinite cylindrical ducts, while Ingard [3] investigated sound radiation from a piston mounted at the open end of a finite cylindrical duct, incorporating both the pressure field and higher-order modes. This paper models a duct system where the inlet and outlet are connected to elastic cylindrical shells, and the central region contains membranes. To address the resulting boundary value problem, Mode-Matching (MM) and Galerkin techniques are employed. The MM method has previously been used by Hassan *et al*. [4, 5] to address complex waveguides with structural discontinuities. Nawaz *et al*. [6] applied it to study scattering from membrane discs and step discontinuities, and Satti *et al*. [7] examined similar effects from rigid plates. Related MM studies for rectangular and circular geometries can be found in [8–12], although comprehensive studies involving flexible shells and membranes remain limited.

The Mode Matching (MM) method is a well-established technique for solving differential equations—such as the Laplace and Helmholtz equations—subject to a variety of boundary conditions, including Dirichlet, Neumann, Robin, and more complex types. This approach involves partitioning the domain into subregions, expressing the acoustic field in each segment as a series of eigenfunctions, and enforcing boundary and interface conditions to derive a tractable system of linear algebraic equations. When the system is governed by the Helmholtz equation with rigid, soft, or impedance-type boundaries, the associated eigenfunctions are typically orthogonal. However, in cases involving higher-order boundary conditions—such as those arising from membrane, plate, or shell dynamics—the eigenfunctions become non-orthogonal and satisfy generalized orthogonality relations. This distinction is particularly relevant in systems exhibiting fluid-structure interaction [13–19]. To address such complexities, Lawrie and Afzal [20] extended the MM framework using a tailored Galerkin approach capable of handling higher-order continuity conditions in membrane-coupled waveguides. Building on this, Afzal and Shafique [21] applied the method to investigate acoustic attenuation in flexible chambers with flanged ends. Despite these advancements, further research is needed to adapt MM techniques to cylindrical shells with embedded membranes and abrupt geometric transitions. In particular, developing generalized orthogonality relations and robust strategies for managing complex interface dynamics remains an open area of investigation.

This study focuses on the dynamic behavior of a flexible shell within a segmented cylindrical waveguide. The governing system is derived from the Helmholtz and Donnell-Mushtari equations, along with boundary conditions as described by Junger and Feit [22] and Lissa [23]. The solution framework involves matching pressure and velocity fields across interfaces and establishing generalized orthogonality conditions. Although MM has been extensively applied to rectangular domains [24–28], its application to flexible circular cylindrical shells with embedded membranes remains underexplored. The elastic shells and membranes are connected via rings that define the physical boundary conditions, typically assumed to be clamped at the connections. Eigenfunction expansions and their generalized properties are used to reduce the governing system to a solvable linear algebraic form. Convergence is ensured using generalized Fourier series techniques, as outlined in [29].

The structure of the article is as follows: Sect 2 outlines the mathematical formulation, including the coupled wave and shell equations. Sect 3 explains the Galerkin approach to model the membrane dynamics, while Sect 4 presents the Mode-Matching (MM) solution of the problem. Sect 5 discusses the energy flux identities along with numerical results, and Sect 6 concludes with a summary of findings.

## 2 Problem formulation

**Fig 1:**
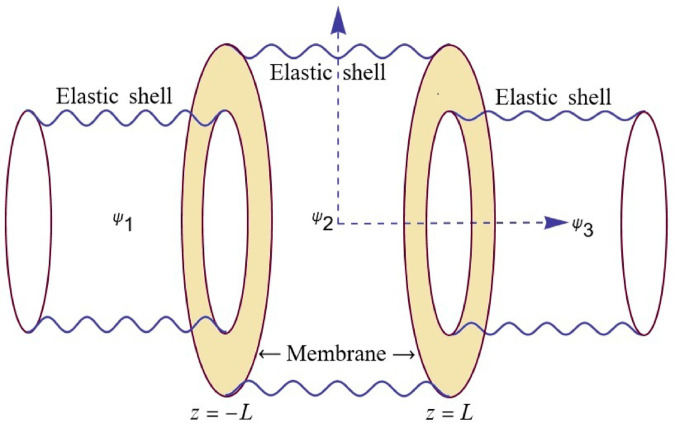
The physical configuration of the problem.

We analyze a cylindrical waveguide divided into three distinct regions, interconnected by flexible shells and elastic membranes. The waveguide is filled with a compressible fluid characterized by sound speed *c* and density $\rho_{0}$. The inlet and outlet regions, located at $\bar{z}<-\bar{L}$ and $\bar{z}>\bar{L}$, respectively, have a radius *r* = *a*. The central region, spanning from *z* = −*L* to *z* = *L*, is bounded by flexible shells with a larger radius *r* = *b*. Elastic membranes connect these regions, facilitating wave transmission across the waveguide. The behavior of the system is governed by the interaction between the propagating acoustic waves, the flexible boundaries, and the varying radii. All dimensional quantities are denoted using an overbar. The physical configuration of the problem is illustrated in Fig 1. Let the time-dependent fluid potential be expressed as $\bar{\Psi }(\bar{r},\bar{z})=e^{-i\omega \bar{t}}\bar{\psi }(\bar{r},\bar{z})$, where ω=ck is the angular frequency, *t* denotes time, and *k* is the wavenumber in the fluid. The dynamics of the flexible shell, extensively studied by Junger and Feit [22], are modeled using the Donnell-Mushtari shell theory as presented by Leissa [23]. This theory is based on the assumptions that the shell displacements are small relative to its thickness, and that the thickness is small compared to the shell’s radius. The Donnell-Mushtari equations for shells with harmonic time dependence, exists when $\bar{r}=\{\bar{a},\bar{b}\}$. For $r=\bar{a}$ the equations are:

$$\frac{\partial^{2}\bar{u}}{\partial {\bar{z}}^{2}}+\frac{1-\nu }{2{\bar{a}}^{2}}\frac{\partial^{2}\bar{u}}{\partial {\bar{\theta }}^{2}}+\frac{1+\nu }{2\bar{a}}\frac{\partial^{2}\bar{v}}{\partial \bar{z}\partial \bar{\theta }}+\frac{\nu }{\bar{a}}\frac{\partial \bar{w}}{\partial \bar{z}}+\frac{\omega^{2}\bar{u}}{c_{s}^{2}}=0,$$ (1)

$$\frac{1+\nu }{2\bar{a}}\frac{\partial^{2}\bar{u}}{\partial \bar{z}\partial \bar{\theta }}+\frac{1-\nu }{2}\frac{\partial^{2}\bar{v}}{\partial {\bar{z}}^{2}}+\frac{1}{{\bar{a}}^{2}}\frac{\partial^{2}\bar{v}}{\partial {\bar{\theta }}^{2}}+\frac{1}{{\bar{a}}^{2}}\frac{\partial \bar{w}}{\partial \bar{\theta }}+\frac{\omega^{2}\bar{v}}{c_{s}^{2}}=0,$$ (2)

$$\frac{\nu }{\bar{a}}\frac{\partial \bar{u}}{\partial \bar{z}}+\frac{1}{{\bar{a}}^{2}}\frac{\partial \bar{v}}{\partial \bar{\theta }}+\frac{\bar{w}}{{\bar{a}}^{2}}+\frac{{\bar{h}}^{2}}{12}\frac{\partial^{4}\bar{w}}{\partial {\bar{z}}^{4}}+\frac{2{\bar{h}}^{2}}{12{\bar{a}}^{2}}\frac{\partial^{4}\bar{w}}{\partial {\bar{z}}^{2}\partial {\bar{\theta }}^{2}}+\frac{{\bar{h}}^{2}}{12{\bar{a}}^{4}}\frac{\partial^{4}\bar{w}}{\partial {\bar{\theta }}^{4}}-\frac{\omega^{2}\bar{w}}{c_{s}^{2}}-\frac{\bar{p}(\bar{a},\bar{z})}{c_{s}^{2}\rho_{s}\bar{h}}=0.$$ (3)

In these equations, $\bar{u}$, $\bar{v}$, and $\bar{w}$ represent the longitudinal, circumferential, and transverse displacements, respectively. The shell has thickness *h*, density $\rho_{s}$, and wave speed $c_{s}=\sqrt{E/((1-\nu^{2})\rho_{s})}$, where *E* and ν are the Young’s modulus and Poisson’s ratio, respectively. The fluid pressure acting on the shell is denoted by $\bar{p}$. Likewise, equations for the central region can be achieved by replacing $\bar{r}=\bar{b}$ in Eqs (1)–(3).

The finite ends of the flexible shells are subject to clamped boundary conditions. A clamped condition implies that all displacement components are constrained to zero, meaning there is no movement in any direction at the boundary. In a dimensional formulation, the clamped condition is represented by the following expression:

$$\bar{u}(\bar{r},\bar{z})=\bar{v}(\bar{r},\bar{z})=\frac{\partial \bar{\psi }}{\partial \bar{r}}(\bar{r},\bar{z})=\frac{\partial^{2}\bar{\psi }}{\partial \bar{r}\partial \bar{z}}(\bar{r},\bar{z})=0\;\text{at}\;\bar{z}=\pm \bar{L},\;\bar{r}=\{\bar{a},\bar{b}\}.$$ (4)

The acoustic field inside a cylindrical waveguide, described using cylindrical coordinates $\left(\bar{r},\bar{\theta },\bar{z}\right)$, is governed by the Helmholtz equation for the fluid potential $\bar{\psi }$ in a homogeneous medium (see [23]), which can be written as:

$$\left\{\frac{\partial^{2}}{\partial {\bar{r}}^{2}}+\frac{1}{\bar{r}}\frac{\partial }{\partial \bar{r}}+\frac{\partial^{2}}{\partial {\bar{z}}^{2}}+k^{2}\right\}\bar{\psi }=0.$$ (5)

### 2.1 Eigenfunction expansions with non-dimensional setting

To nondimensionalize the governing equations, we introduce a length scale ${\text{k}}^{-1}$ and a time scale $\omega^{-1}$, leading to the transformations $k\bar{r}=r$, $k\bar{z}=z$, and $k^{2}\bar{\psi }=\omega \psi$. In what follows, nondimensional quantities are denoted using the same symbols as their dimensional counterparts, but without the overbar. The fluid potential ψ(r,z) can be expressed piecewise over the three regions of the waveguide as:

$$\psi \left(r,z\right)=\left\{ \begin{array}{c} \psi_{1}\left(r,z\right),\;\text{in Region-I,}\\ \psi_{2}\left(r,z\right),\;\text{in Region-II,}\\ \psi_{3}\left(r,z\right),\;\text{in Region-III,} \end{array}\right.$$ (6)

where each $\psi_{j}$ for j=1,2,3 satisfies the nondimensional Helmholtz equation in its respective region:

$$\left\{\frac{\partial^{2}}{\partial r^{2}}+\frac{1}{r}\frac{\partial }{\partial r}+\frac{\partial^{2}}{\partial z^{2}}+1\right\}\psi_{j}=0.$$ (7)

#### 2.1.1 Regions I and III.

The dimensionless equations of motion for the flexible shells in the waveguide are derived under the assumption of axisymmetric vibration and small displacements. These equations follow from the Donnell-Mushtari shell theory and apply to regions bounded by flexible shells at *r* = *a*. The dimensionless form can be expressed as:

$$\frac{\partial^{2}u}{\partial z^{2}}+\frac{i\nu }{a}\frac{\partial^{2}\psi }{\partial r\partial z}+\beta^{2}u=0,$$ (8)

$$\frac{1-\nu }{2}\frac{\partial^{2}v}{\partial z^{2}}+\beta^{2}\nu =0,$$ (9)

$$-i\nu \frac{\partial u}{\partial z}+\frac{\partial \psi }{\partial r}+\frac{1}{\Gamma }\frac{\partial^{5}\psi }{\partial r\partial z^{4}}-a^{2}\beta^{2}\frac{\partial \psi }{\partial r}-\frac{a^{2}\beta^{2}\rho }{\rho_{s}hk}\psi =0.$$ (10)

Here, $\beta =\omega /(c_{s}k)$ and $\Gamma =12/(k^{2}h^{2}a^{2})$ are dimensionless parameters. The velocity potential in the duct regions I and III is expressed using eigenfunction expansions:

$$\psi_{1}(r,z)=F_{\ell }J_{0}(\xi_{\ell }r)e^{i\eta_{\ell }(z+L)}+\sum_{n=0}^{\infty }A_{n}J_{0}(\xi_{n}r)e^{-i\eta_{n}(z+L)},$$ (11)

Using this potential, the shell’s displacement components in region I can be expressed as:

$$u_{1}(a,z)=-\frac{\nu F_{\ell }\eta_{\ell }J_{0}^{\prime }(\xi_{\ell }a)e^{i\eta_{\ell }(z+L)}}{(\eta_{\ell }^{2}-\beta^{2})a}+\sum_{n=0}^{\infty }\frac{\nu A_{n}\eta_{n}J_{0}^{\prime }(\xi_{n}a)e^{-i\eta_{n}(z+L)}}{(\eta_{n}^{2}-\beta^{2})a},$$ (12)

$$w_{1}(a,z)=F_{\ell }J_{0}^{\prime }(\xi_{\ell }a)e^{i\eta_{\ell }(z+L)}+\sum_{n=0}^{\infty }A_{n}J_{0}^{\prime }(\xi_{n}a)e^{-i\eta_{n}(z+L)}.$$ (13)

Similarly, for region III, the velocity potential and displacements are:

$$\psi_{3}(r,z)=\sum_{n=0}^{\infty }D_{n}J_{0}(\xi_{n}r)e^{i\eta_{n}(z-L)},$$ (14)

$$u_{3}(a,z)=-\sum_{n=0}^{\infty }\frac{\nu D_{n}\eta_{n}J_{0}^{\prime }(\xi_{n}a)e^{i\eta_{n}(z-L)}}{(\eta_{n}^{2}-\beta^{2})a},$$ (15)

$$w_{3}(a,z)=-\sum_{n=0}^{\infty }D_{n}J_{0}^{\prime }(\xi_{n}a)e^{i\eta_{n}(z-L)}.$$ (16)

The modal wavenumbers $\eta_{n}=\sqrt{1-\xi_{n}^{2}}$ are defined via the eigenvalues $\xi_{n}$, which satisfy the dispersion relation obtained from solving the Donnell-Mushtari equations:

$$-\Gamma \nu^{2}(1-\xi^{2})\xi J_{1}(\xi a)+\left[(1-\xi^{2})-\beta^{2}\right]\left[(1-\xi^{2}{)}^{2}-\mu^{4}\right]\xi J_{1}(\xi a)\;+\left[(1-\xi^{2})-\beta^{2}\right]\alpha J_{0}(\xi a)=0,$$ (17)

where $\alpha =\frac{12\beta^{2}\rho_{0}}{\rho_{s}h^{3}k^{3}}$ is the fluid loading parameter and $\mu^{4}=\Gamma (a^{2}\beta^{2}\;-\;1)$ is related to the shell wavenumber. As shown in [7, 9], the eigenfunctions $\{J_{0}(\xi_{n}r){\}}_{n=0}^{\infty }$ form a generalized (non-orthogonal) system that satisfies the following orthogonality-like relation:

$$\frac{\alpha }{a}\int_{0}^{a}J_{0}(\xi_{m}r)J_{0}(\xi_{n}r)r\;dr=\delta_{mn}X_{n}-\left\{\frac{\Gamma \nu^{2}\beta^{2}}{\left(\eta_{n}^{2}-\beta^{2})(\eta_{m}^{2}-\beta^{2}\right)}+2-\xi_{m}^{2}-\xi_{n}^{2}\right\}J_{0}^{\prime }(\xi_{m}a)J_{0}^{\prime }(\xi_{n}a),$$ (18)

where


$$X_{n}=\frac{\alpha a}{2}\left[J_{0}^{2}(\xi_{n}a)+J_{1}^{2}(\xi_{n}a)\right]+\left(\frac{\Gamma \nu^{2}\beta^{2}}{(\eta_{n}^{2}-\beta^{2}{)}^{2}}+2\eta_{n}^{2}\right){\left[J_{0}^{\prime }(\xi_{n}a)\right]}^{2}.$$

In Eqs (11)–(13), the first term on the right-hand side represents the incident wave. This is assumed by letting $A_{n}=F_{\ell }\delta_{n\ell }$, where ℓ=0 or 1 denotes the fundamental or next higher mode under consideration. The corresponding amplitude is given by


$$F_{\ell }=\sqrt{\frac{\alpha }{a\eta_{\ell }X_{\ell }}}.$$

#### 2.1.2 Region II.

The velocity potential in the expansion chamber (Region II) results from both the waves reflected at the second junction and those transmitted through the first junction. As in the previous section, the Helmholtz equation is solved subject to appropriate boundary conditions. The resulting expression for the velocity potential $\psi_{2}$ at *r* = *b* is given by:

$$\psi_{2}(r,z)=\sum_{n=0}^{\infty }B_{n}J_{0}(\gamma_{n}r)e^{is_{n}z}+\sum_{n=0}^{\infty }C_{n}J_{0}(\gamma_{n}r)e^{-is_{n}z},$$ (19)

The associated longitudinal and radial displacements of the flexible shell in Region II can similarly be expressed as eigenfunction expansions:

$$u_{2}(b,z)=-\sum_{n=0}^{\infty }\frac{\nu B_{n}s_{n}J_{0}^{\prime }(\gamma_{n}b)e^{is_{n}(z+L)}}{(s_{n}^{2}-\beta^{2})b}+\sum_{n=0}^{\infty }\frac{\nu C_{n}s_{n}J_{0}^{\prime }(\gamma_{n}b)e^{-is_{n}(z+L)}}{(s_{n}^{2}-\beta^{2})b},$$ (20)

$$w_{2}(b,z)=\sum_{n=0}^{\infty }B_{n}J_{0}^{\prime }(\gamma_{n}b)e^{is_{n}(z+L)}+\sum_{n=0}^{\infty }C_{n}J_{0}^{\prime }(\gamma_{n}b)e^{-is_{n}(z+L)}.$$ (21)

Each mode in Region II is characterized by a wavenumber $s_{n}=\sqrt{1-\gamma_{n}^{2}}$, where $\gamma_{n}$ is the eigenvalue associated with the mode *n*. These eigenvalues $\gamma_{n}$ are determined by solving the characteristic equation derived from the Donnell-Mushtari shell equations:

$$-\Gamma \nu^{2}(1-\gamma^{2})\gamma J_{1}(\gamma b)+\left[(1-\gamma^{2})-\beta^{2}\right]\left[(1-\gamma^{2}{)}^{2}-\mu^{4}\right]\gamma J_{1}(\gamma b)\;\;+\left[(1-\gamma^{2})-\beta^{2}\right]\alpha J_{0}(\gamma b)=0.$$ (22)

In this context, the parameters $\alpha =\frac{12\beta^{2}\rho_{0}}{\rho_{s}h^{3}k^{3}}$ and $\mu^{4}=\Gamma (\beta^{2}\;-\;1)/b^{2}$ represent the fluid loading parameter and the shell wavenumber, respectively. For all n=0,1,2,…, the eigenfunctions $J_{0}(\gamma_{n}r)$ are non-orthogonal but satisfy a generalized orthogonality relation of the form:

$$\frac{\alpha }{b}\int_{0}^{b}J_{0}(\gamma_{m}r)J_{0}(\gamma_{n}r)r\;dr=\delta_{mn}Y_{n}-\left\{\frac{\Gamma \nu^{2}\beta^{2}}{\left(s_{n}^{2}-\beta^{2})(s_{m}^{2}-\beta^{2}\right)}+2-\gamma_{m}^{2}-\gamma_{n}^{2}\right\}J_{0}^{\prime }(\gamma_{m}b)J_{0}^{\prime }(\gamma_{n}b),$$ (23)

where


$$Y_{n}=\frac{\alpha b}{2}\left[J_{0}^{2}(\gamma_{n}b)+J_{1}^{2}(\gamma_{n}b)\right]+\left(\frac{\Gamma \nu^{2}\beta^{2}}{(s_{n}^{2}-\beta^{2}{)}^{2}}+2s_{n}^{2}\right){\left[J_{0}^{\prime }(\gamma_{n}b)\right]}^{2}.$$

## 3 Galerkin formulation

At the interfaces of the system, elastic membrane discs are present, and their dynamic behavior can be described by the following dimensional form of the governing equation [22]:

$$T{\bar{\nabla }}^{2}\bar{Q}+\omega^{2}\rho_{m}\bar{Q}=[\bar{p}{]}_{-}^{+},$$ (24)

where *T* denotes the membrane tension, $\rho_{m}$ is the membrane density, and $\bar{Q}(r)$ represents the dimensional membrane displacement. The membranes are assumed to be clamped at the end rings, satisfying the boundary conditions:

$$\bar{Q}(\bar{a})=\bar{Q}(\bar{b})=0,\;\bar{z}=\pm \bar{L}.$$ (25)

The equations are non-dimensionalized using the characteristic length scale ${\text{k}}^{-1}$. At positions *z* = −*L* and *z* = *L*, the dimensionless membrane displacements are defined as $e_{1}=k{\bar{Q}}_{1}$ and $e_{2}=k{\bar{Q}}_{2}$, respectively. The governing equations for the membrane dynamics become:

$$\left\{\frac{\partial^{2}}{\partial r^{2}}+\frac{1}{r}\frac{\partial }{\partial r}+\mu_{m}^{2}\right\}e_{1}=\alpha_{m}\psi_{2},\;z=-L,$$ (26)

$$\left\{\frac{\partial^{2}}{\partial r^{2}}+\frac{1}{r}\frac{\partial }{\partial r}+\mu_{m}^{2}\right\}e_{2}=\alpha_{m}\psi_{2},\;z=L,$$ (27)

where $\mu_{m}=c/c_{m}$ is the dimensionless membrane wavenumber, and $\alpha_{m}=c^{2}\rho_{0}/(kT)$ characterizes the fluid loading effect. The membranes are assumed to be clamped to the supporting rim, resulting in fixed edge conditions:

$$e_{j}(r)=0,\;r\in \{a,b\},\;z=\pm L,\;\text{for}\;j=1,2.$$ (28)

To solve the eigenvalue problem posed by the differential equations with the above boundary conditions, the Galerkin method is applied. The membrane displacements are expanded in terms of eigenfunctions as:

$$e_{1}=\sum_{n=1}^{\infty }H_{1n}I(\lambda_{n}r),$$ (29)

$$e_{2}=\sum_{n=1}^{\infty }H_{2n}I(\lambda_{n}r),$$ (30)

where the eigenfunctions $I(\lambda_{n}r)$ are defined as:


$$I(\lambda_{n}r)=J_{0}(\lambda_{n}r)N_{0}(\lambda_{n}a)-J_{0}(\lambda_{n}a)N_{0}(\lambda_{n}r),$$

and the eigenvalues $\lambda_{n}$ are the roots of the characteristic equation $I(\lambda_{n}b)=0$. These eigenfunctions satisfy the following orthogonality relation:

$$\int_{a}^{b}I(\lambda_{n}r)I(\lambda_{m}r)r\;dr=E_{n}\delta_{mn},\;\text{with}\;E_{n}=\int_{a}^{b}I^{2}(\lambda_{n}r)r\;dr.$$ (31)

The coefficients *H*_*jn*_ for j=1,2 remain to be determined and will be obtained by enforcing the membrane interface conditions presented in Eqs (26) and (27).

## 4 Mode matching formulation

To determine the unknown coefficients, the *mode-matching method* is employed, as detailed in [18–21]. This method is fundamental for evaluating the modal amplitudes $\left\{A_{n},B_{n},C_{n},D_{n}\right\}$ and $\{H_{1n},H_{2n}\},$ and it relies on the continuity of pressure, velocity, and membrane displacement across the interfaces. The matching conditions at the boundaries z=±L ensure continuity of the acoustic field between adjacent regions. These conditions relate the coefficients from the eigenfunction expansions in each region:

$$\psi_{1}(r,z)=\psi_{2}(r,z),\;z=-L,\;0\leq r\leq a,$$ (32)

$$\psi_{2}(r,z)=\psi_{3}(r,z),\;z=L,\;0\leq r\leq a,$$ (33)

$$\frac{\partial \psi_{2}}{\partial z}=\left\{ \begin{array}{cc} \frac{\partial \psi_{1}}{\partial z}, & 0\leq r\leq a,\;z=-L,\\ e_{1}, & a\leq r\leq b,\;z=-L, \end{array}\right.$$ (34)

$$\frac{\partial \psi_{3}}{\partial z}=\left\{ \begin{array}{cc} \frac{\partial \psi_{2}}{\partial z}, & 0\leq r\leq a,\;z=L,\\ e_{2}, & a\leq r\leq b,\;z=L. \end{array}\right.$$ (35)

Note that the assumed configuration is symmetric about *z* = 0. Before using the expansions into matching conditions, first we split the problem into symmetric and anti-symmetric sub-problems by using the symmetry of the structure. The adopted approach is mathematically simpler and yield the same results These sub-problems are explained subsequently.

### 4.1 Symmetric case

We considered $B_{n}=C_{n}$ for the symmetric case with unknown amplitudes $B_{n}^{s}$ and $C_{n}^{s}$ and took the specific values of variables in the symmetric setting, indicated by the superscript “$s^{\prime \prime }$. After simplification, we get Eqs (11), (19) and (29) as

$$\psi_{1}^{s}(r,z)=F_{\ell }J_{0}(\xi_{\ell },r)e^{i\eta_{\ell }(z+L)}+\sum_{n=0}^{\infty }A_{n}^{s}J_{0}(\xi_{n},r)e^{-i\eta_{n}(z+L)},$$ (36)

$$\psi_{2}^{s}(r,z)=2\sum_{n=0}^{\infty }B_{n}^{s}J_{0}(\gamma_{n},r)\cos(s_{n}z),$$ (37)

$$e_{1}^{s}=\sum_{n=1}^{\infty }H_{1n}^{s}I(\lambda_{n}r).$$ (38)

By using (37) and (38) into Eq (26) at *z* = −*L*, after some mathematical rearrangements we get $H_{1n}^{s}$ for the symmetric case as

$$H_{1m}^{s}=\frac{\;\alpha_{m}}{E_{m}\;\Lambda_{m}}\left\{2\sum_{n=0}^{\infty }B_{n}^{s}\;\cos(s_{n}L){△}_{mn}\;\right\},$$ (39)

where


$$\Lambda_{m}=\mu_{m}^{2}-\lambda_{m}^{2}\;\;\;{△}_{mn}=\int_{a}^{b}I(\lambda_{m}r)J_{0}(\gamma_{n},r)rdr,$$

On multiplying the matching condition (32) with $\frac{\alpha }{a}J_{0}(\xi_{m},r)$ and then integrating 0 to *a*

$$\frac{\alpha }{a}\int_{0}^{a}J_{0}(\xi_{m},r)\psi_{1}^{s}(r,z)rdr=\frac{\alpha }{a}\int_{0}^{a}J_{0}(\xi_{m},r)\psi_{2}^{s}(r,z)rdr.$$ (40)

By using Eqs (36) and (37) into (40) and using orthognality relation (17), we obtain $A_{n}^{s}$ after simplification as

$$A_{m}^{s}=-F_{\ell }\delta_{m\ell }+\frac{J_{0}^{\prime }(\xi_{m},a)}{X_{m}}\left(\frac{N_{0}^{s}}{\eta_{m}^{2}-\beta^{2}}+(2-\xi_{m}^{2})N_{1}^{s}-N_{2}^{s}\right)+\frac{2\alpha }{a}\sum_{n=0}^{\infty }\frac{B_{n}^{s}R_{mn}\cos(s_{n}L)}{X_{m}}.$$ (41)

where


$$R_{mn}=\int_{0}^{a}J_{0}(\xi_{m},r)J_{0}(\gamma_{n},r)rdr,$$

$$N_{0}^{s}=\Gamma \nu^{2}\beta^{2}\left\{\frac{F_{\ell }J_{0}^{\prime }(\xi_{\ell },a)}{\eta_{\ell }^{2}-\beta^{2}}+\sum_{n=0}^{\infty }\frac{A_{n}^{s}J_{0}^{\prime }(\xi_{n},a)}{\eta_{n}^{2}-\beta^{2}}\right\}.$$

$$N_{1}^{s}=F_{\ell }J_{0}^{\prime }(\xi_{\ell },a)+\sum_{n=0}^{\infty }A_{n}^{s}J_{0}^{\prime }(\gamma_{n},a)=\frac{\partial \psi_{1}^{s}}{\partial r}(a,-L).$$

$$N_{2}^{s}=F_{\ell }\tau_{\ell }^{2}J_{0}^{\prime }(\xi_{\ell },a)+\sum_{n=0}^{\infty }A_{n}^{s}\xi_{n}^{2}J_{0}^{\prime }(\xi_{n},a).$$

Here $N_{0}^{s}$, $N_{1}^{s}$ and $N_{2}^{s}$ are unknown constants. On performing multiplication with $\frac{\alpha }{b}J_{0}(\gamma_{m},r)$ to matching condition (34) and integrating “0” to “b”

$$\frac{\alpha }{b}\int_{0}^{b}J_{0}(\gamma_{m},b)\frac{\partial \psi_{2}^{s}}{\partial z}rdr=\frac{\alpha }{b}\int_{0}^{a}J_{0}(\gamma_{m},b)\frac{\partial \psi_{1}^{s}}{\partial z}rdr+\frac{\alpha }{b}\int_{a}^{b}J_{0}(\gamma_{m},b)e_{1}^{s}(r)rdr.$$ (42)

Substituting Eqs (36), (37) and (38) into (42), after simplification with the aid of orthonality relation (23), we get

$$B_{m}^{s}=\frac{J_{0}^{\prime }(\gamma_{m},b)}{s_{m}\sin(s_{m},L)Y_{m}}\left(\frac{M_{0}^{s}}{s_{m}^{2}-\beta^{2}}+(2-\gamma_{m}^{2})M_{1}^{s}-M_{2}^{s}\right)+\frac{i\alpha \eta_{\ell }F_{\ell }R_{\ell m}}{2bs_{m}\sin(s_{m},L)Y_{m}}\;\;+\frac{\alpha }{2bs_{m}\sin(s_{m},L)Y_{m}}\sum_{n=0}^{\infty }(\Delta_{nm}H_{1n}^{s}-i\eta_{n}A_{n}^{s}R_{nm}).$$ (43)

where


$$M_{0}^{s}=\Gamma \nu^{2}\beta^{2}\left\{\sum_{n=0}^{\infty }\frac{s_{n}B_{n}^{s}\sin(s_{n},L)J_{0}^{\prime }(\gamma_{n},b)}{s_{n}^{2}-\beta^{2}}\right\}=\Gamma \nu^{2}\beta^{2}u(b,-L).$$

$$M_{1}^{s}=\sum_{n=0}^{\infty }s_{n}B_{n}^{s}\sin(s_{n},L)J_{0}^{\prime }(\gamma_{n},b)=\frac{\partial }{\partial r}(\frac{\partial \psi_{1}^{s}}{\partial z})(b,-L).$$

$$M_{2}^{s}=\sum_{n=0}^{\infty }s_{n}\sin(s_{n},L)B_{n}^{s}\gamma_{n}^{2}J_{0}^{\prime }(\gamma_{n},b).$$

It is important to note that the constants $N_{0}^{s}$, $N_{1}^{s}$, $N_{2}^{s}$, $M_{0}^{s}$, $M_{1}^{s}$, and $M_{2}^{s}$ appearing in Eqs (41) and (43) remain undetermined. These constants can be evaluated by applying the clamped boundary conditions. Specifically, under these conditions, $N_{1}^{s}=0$, while $N_{0}^{s}$ and $N_{2}^{s}$ are non-zero. Conversely, $M_{0}^{s}=0$ and $M_{1}^{s}=0$, whereas $M_{2}^{s}\neq 0$.

To determine the coefficients $N_{0}^{s}$ and $N_{2}^{s}$, we substitute Eq (36) into the clamped boundary condition given by Eq (4), which leads to the following constraint relations:

$$\sum_{n=0}^{\infty }\frac{A_{n}^{s}\eta_{n}J_{0}^{\prime }(\xi_{n},a)}{\eta_{n}^{2}-\beta^{2}}=\frac{F_{\ell }\eta_{\ell }J_{0}^{\prime }(\xi_{\ell },a)}{\eta_{\ell }-\beta^{2}},$$ (44)

$$\sum_{n=0}^{\infty }\eta_{n}A_{n}^{s}J_{0}^{\prime }(\xi_{n},a)=F_{\ell }\eta_{\ell }J_{0}^{\prime }(\xi_{\ell },a).$$ (45)

By multiplying Eq (41) with the functions $\sum_{m=0}^{\infty }\frac{\eta_{m}J_{0}^{\prime }(\xi_{m},a)}{\eta_{m}^{2}-\beta^{2}}$ and $\sum_{m=0}^{\infty }\eta_{m}J_{0}^{\prime }(\xi_{m},a)$, and substituting the constraint conditions (44) and (45), respectively, we obtain:

$$\Sigma_{11}N_{0}^{s}-\Sigma_{12}N_{2}^{s}=\frac{2\eta_{\ell }F_{\ell }J_{0}^{\prime }(\xi_{\ell },a)}{\eta_{\ell }^{2}-\beta^{2}}-\frac{2\alpha }{a}\sum_{n=0}^{\infty }\sum_{m=0}^{\infty }\frac{B_{n}^{s}\eta_{m}J_{0}^{\prime }(\xi_{m},a)R_{mn}\cos(s_{n}L)}{(\eta_{m}^{2}-\beta^{2})X_{m}},$$ (46)

$$\Sigma_{21}N_{0}^{s}-\Sigma_{22}N_{2}^{s}=2F_{\ell }\eta_{\ell }J_{0}^{\prime }(\xi_{\ell },a)-\frac{2\alpha }{a}\sum_{n=0}^{\infty }\sum_{m=0}^{\infty }\frac{B_{n}^{s}\eta_{m}J_{0}^{\prime }(\xi_{m},a)R_{mn}\cos(s_{n}L)}{X_{m}}.$$ (47)

where the coefficients are defined as:


$$\Sigma_{11}=\sum_{m=0}^{\infty }\frac{\eta_{m}(J_{0}^{\prime }(\xi_{m},a){)}^{2}}{(\eta_{m}^{2}-\beta^{2}{)}^{2}X_{m}},\;\Sigma_{12}=\sum_{m=0}^{\infty }\frac{\eta_{m}(J_{0}^{\prime }(\xi_{m},a){)}^{2}}{(\eta_{m}^{2}-\beta^{2})X_{m}},$$

$$\Sigma_{21}=\sum_{m=0}^{\infty }\frac{\eta_{m}(J_{0}^{\prime }(\xi_{m},a){)}^{2}}{(\eta_{m}^{2}-\beta^{2})X_{m}},\;\Sigma_{22}=\sum_{m=0}^{\infty }\frac{\eta_{m}(J_{0}^{\prime }(\xi_{m},a){)}^{2}}{X_{m}}.$$

By solving the linear system represented by Eqs (46) and (47) simultaneously, the values of $N_{0}^{s}$ and $N_{2}^{s}$ can be determined.

To calculate $M_{2}^{s}$, we substitute Eq (37) into the clamped boundary condition (4), leading to the constraint:

$$\sum_{n=0}^{\infty }B_{n}^{s}\cos(s_{n}L)J_{0}^{\prime }(\gamma_{n},b)=0.$$ (48)

Multiplying (43) by $\sum_{m=0}^{\infty }J_{0}^{\prime }(\gamma_{m},b)\cos(s_{m}L)$, and applying the constraint from (48), we simplify to obtain:

$$M_{2}^{s}=\frac{1}{S_{1}^{s}}\{\frac{i\alpha \eta_{\ell }F_{\ell }}{2b}\sum_{m=0}^{\infty }\frac{R_{\ell m}\cos(s_{m}L)J_{0}^{\prime }(\gamma_{m},b)}{s_{m}\sin(s_{m}L)Y_{m}}\;\;+\frac{\alpha }{2b}\sum_{n=0}^{\infty }\sum_{m=0}^{\infty }\frac{\cos(s_{m}L)J_{0}^{\prime }(\gamma_{m},b)}{s_{m}\sin(s_{m}L)Y_{m}}\left(\Delta_{nm}H_{1n}^{s}-i\eta_{n}A_{n}^{s}R_{nm}\right)\},$$ (49)

where


$$S_{1}^{s}=\sum_{m=0}^{\infty }\frac{\cos(s_{m}L)(J_{0}^{\prime }(\gamma_{m},b){)}^{2}}{s_{m}\sin(s_{m}L)Y_{m}}.$$

Finally, Eqs (41) and (43) can be used to generate systems of linear algebraic equations for indices m=n=0,1,2,…,N, based on the clamped edge conditions.

### 4.2 Anti-symmetric case

For the anti-symmetric case, we set $B_{n}=-C_{n}$ and adopted specific values of variables appropriate for this configuration, denoted by the superscript “*a*”. After simplification, we obtain the following expressions from Eqs (11) and (19):

$$\psi_{1}^{a}(r,z)=F_{\ell }J_{0}(\xi_{\ell },r)e^{i\eta_{\ell }(z+L)}+\sum_{n=0}^{\infty }A_{n}^{a}J_{0}(\xi_{n},r)e^{-i\eta_{n}(z+L)},$$ (50)

$$\psi_{2}^{a}(r,z)=2i\sum_{n=0}^{\infty }B_{n}^{a}J_{0}(\gamma_{n},r)\sin(s_{n}z),$$ (51)

$$e_{1}^{a}(r)=\sum_{n=1}^{\infty }H_{1n}^{a}I(\lambda_{n}r).$$ (52)

Substituting Eqs (37) and (38) into (26) at *z* = −*L*, and performing algebraic manipulation, the coefficient $H_{1m}^{a}$ in the anti-symmetric case is obtained as:

$$H_{1m}^{a}=-\frac{2i\alpha_{m}}{O_{m}\Lambda_{m}}\left\{\sum_{n=0}^{\infty }B_{n}^{a}\sin(s_{n}L)\Delta_{mn}\right\}.$$ (53)

Multiplying the matching condition (32) by $\frac{\alpha }{a}J_{0}(\xi_{m},r)$ and integrating from 0 to *a* gives:

$$\frac{\alpha }{a}\int_{0}^{a}J_{0}(\xi_{m},r)\psi_{1}^{a}(r,z)r\;dr=\frac{\alpha }{a}\int_{0}^{a}J_{0}(\xi_{m},r)\psi_{2}^{a}(r,z)r\;dr.$$ (54)

Using (50) and (51) in (54), along with the orthogonality relation (17), we obtain the coefficient $A_{m}^{a}$:

$$A_{m}^{a}=-F_{\ell }\delta_{m\ell }+\frac{J_{0}^{\prime }(\xi_{m},a)}{X_{m}}\left(\frac{N_{0}^{a}}{\eta_{m}^{2}-\beta^{2}}+(2-\xi_{m}^{2})N_{1}^{a}-N_{2}^{a}\right)-\frac{2i\alpha }{a}\sum_{n=0}^{\infty }\frac{B_{n}^{a}R_{mn}\sin(s_{n}L)}{X_{m}}.$$ (55)

Here, the constants are defined as:


$$N_{0}^{a}=\Gamma \nu^{2}\beta^{2}\left\{\frac{F_{\ell }J_{0}^{\prime }(\xi_{\ell },a)}{\eta_{\ell }^{2}-\beta^{2}}+\sum_{n=0}^{\infty }\frac{A_{n}^{a}J_{0}^{\prime }(\xi_{n},a)}{\eta_{n}^{2}-\beta^{2}}\right\},$$

$$N_{1}^{a}=F_{\ell }J_{0}^{\prime }(\xi_{\ell },a)+\sum_{n=0}^{\infty }A_{n}^{a}J_{0}^{\prime }(\gamma_{n},a)=-{\frac{\partial \psi_{1}^{a}}{\partial r}|}_{\left(a,-L\right)},$$

$$N_{2}^{a}=F_{\ell }\xi_{\ell }^{2}J_{0}^{\prime }(\xi_{\ell },a)+\sum_{n=0}^{\infty }A_{n}^{a}\xi_{n}^{2}J_{0}^{\prime }(\xi_{n},a).$$

Next, multiplying the matching condition (34) by $\frac{\alpha }{b}J_{0}(\gamma_{m},r)$ and integrating from 0 to *b* gives:

$$\frac{\alpha }{b}\int_{0}^{b}J_{0}(\gamma_{m},r)\frac{\partial \psi_{2}^{a}}{\partial z}r\;dr=\frac{\alpha }{b}\int_{0}^{a}J_{0}(\gamma_{m},r)\frac{\partial \psi_{1}^{a}}{\partial z}r\;dr+\frac{\alpha }{b}\int_{a}^{b}J_{0}(\gamma_{m},r)e_{1}^{a}(r)r\;dr.$$ (56)

Substituting (50), (51), and (52) into (56) and simplifying with the aid of the orthogonality relation (23), we get:

$$B_{m}^{a}=\frac{J_{0}^{\prime }(\gamma_{m},a)}{s_{m}\cos(s_{m}L)Y_{m}}\left(\frac{M_{0}^{a}}{s_{m}^{2}-\beta^{2}}+(2-\gamma_{m}^{2})M_{1}^{a}-M_{2}^{a}\right)+\frac{\alpha \eta_{\ell }F_{\ell }R_{\ell m}}{2bs_{m}\cos(s_{m}L)Y_{m}}\;\;-\frac{\alpha }{2bs_{m}\cos(s_{m}L)Y_{m}}\sum_{n=0}^{\infty }\left(i\Delta_{nm}H_{1n}^{a}+\eta_{n}A_{n}^{a}R_{nm}\right).$$ (57)

Here, the constants $M_{i}^{a}$ are given by:


$$M_{0}^{a}=\Gamma \nu^{2}\beta^{2}\sum_{n=0}^{\infty }\frac{s_{n}B_{n}^{a}\cos(s_{n}L)J_{0}^{\prime }(\gamma_{n},a)}{s_{n}^{2}-\beta^{2}}=\Gamma \nu^{2}\beta^{2}u(a,-L),$$

$$M_{1}^{a}=\sum_{n=0}^{\infty }s_{n}B_{n}^{a}\cos(s_{n}L)J_{0}^{\prime }(\gamma_{n},a),$$

$$M_{2}^{a}=\sum_{n=0}^{\infty }s_{n}\cos(s_{n}L)B_{n}^{a}\gamma_{n}^{2}J_{0}^{\prime }(\gamma_{n},a).$$

Note that the constants $N_{0}^{a},N_{1}^{a},N_{2}^{a},M_{0}^{a},M_{1}^{a},M_{2}^{a}$ remain undetermined at this stage. These can be evaluated by applying the clamped boundary conditions. For the anti-symmetric case, these conditions imply $N_{1}^{a}=0$, $M_{0}^{a}=0$, and $M_{1}^{a}=0$, while $N_{0}^{a}$, $N_{2}^{a}$, and $M_{2}^{a}$ are non-zero, as inferred from Eqs (55) and (57).

To determine $N_{0}^{a}$ and $N_{2}^{a}$, we apply the clamped boundary condition (4) to (50), which gives:

$$\sum_{n=0}^{\infty }\frac{A_{n}^{a}\eta_{n}J_{0}^{\prime }(\xi_{n},a)}{\eta_{n}^{2}-\beta^{2}}=\frac{F_{\ell }\eta_{\ell }J_{0}^{\prime }(\xi_{\ell },a)}{\eta_{\ell }^{2}-\beta^{2}},$$ (58)

$$\sum_{n=0}^{\infty }\eta_{n}A_{n}^{a}J_{0}^{\prime }(\xi_{n},a)=F_{\ell }\eta_{\ell }J_{0}^{\prime }(\xi_{\ell },a).$$ (59)

Multiplying (55) by $\sum_{m=0}^{\infty }\frac{\eta_{m}J_{0}^{\prime }(\xi_{m},a)}{\eta_{m}^{2}-\beta^{2}}$ and $\sum_{m=0}^{\infty }\eta_{m}J_{0}^{\prime }(\xi_{m},a)$, and applying the constraints (58) and (59), we derive:

$$\Sigma_{11}N_{0}^{a}-\Sigma_{12}N_{2}^{a}=\frac{2\eta_{\ell }F_{\ell }J_{0}^{\prime }(\xi_{\ell },a)}{\eta_{\ell }^{2}-\beta^{2}}+\frac{2i\alpha }{a}\sum_{n=0}^{\infty }\sum_{m=0}^{\infty }\frac{B_{n}^{a}\eta_{m}J_{0}^{\prime }(\xi_{m},a)R_{mn}\sin(s_{n}L)}{(\eta_{m}^{2}-\beta^{2})X_{m}},$$ (60)

$$\Sigma_{21}N_{0}^{a}-\Sigma_{22}N_{2}^{a}=2F_{\ell }\eta_{\ell }J_{0}^{\prime }(\xi_{\ell },a)+\frac{2i\alpha }{a}\sum_{n=0}^{\infty }\sum_{m=0}^{\infty }\frac{B_{n}^{a}\eta_{m}J_{0}^{\prime }(\xi_{m},a)R_{mn}\sin(s_{n}L)}{X_{m}}.$$ (61)

Eqs (60) and (61) form a system of linear equations that can be solved simultaneously to obtain the unknowns $N_{0}^{a}$ and $N_{2}^{a}$.

To determine $M_{2}^{a}$, we apply the clamped boundary condition to (51), which yields:

$$\sum_{n=0}^{\infty }B_{n}^{a}\sin(s_{n}L)J_{0}^{\prime }(\gamma_{n},b)=0.$$ (62)

Multiplying (57) by $\sum_{m=0}^{\infty }J_{0}^{\prime }(\gamma_{m},b)\sin(s_{m}L)$ and simplifying using (62), we find:

$$M_{2}^{a}=\frac{1}{S_{2}^{a}}\{\frac{\alpha \eta_{\ell }F_{\ell }}{2b}\sum_{m=0}^{\infty }\frac{R_{\ell m}\sin(s_{m}L)J_{0}^{\prime }(\gamma_{m},b)}{s_{m}\cos(s_{m}L)Y_{m}}\;\;-\frac{\alpha }{2b}\sum_{n=0}^{\infty }\sum_{m=0}^{\infty }\frac{\sin(s_{m}L)J_{0}^{\prime }(\gamma_{m},b)}{s_{m}\cos(s_{m}L)Y_{m}}\left(i\Delta_{nm}H_{1n}^{a}+\eta_{n}A_{n}^{a}R_{nm}\right)\},$$ (63)

where


$$S_{2}^{a}=\sum_{m=0}^{\infty }\frac{\sin(s_{m}L){\left(J_{0}^{\prime }(\gamma_{m},b)\right)}^{2}}{s_{m}\cos(s_{m}L)Y_{m}}.$$

Based on the clamped boundary conditions, discrete algebraic systems for m=n=0,1,2,…,N can be constructed from Eqs (55) and (57).

## 5 Numerical results and discussions

In the preceding sections, the linear algebraic systems were simplified by truncating the infinite series to *N* terms, with indices m,n=0,1,2,…,N. The resulting finite systems were then solved numerically. This truncation enabled the evaluation of the symmetric and anti-symmetric amplitude components $\left\{A_{m}^{s},A_{m}^{a},B_{m}^{s},B_{m}^{a}\right\}$ and $\left\{H_{1m}^{s},H_{1m}^{a}\right\}$, which characterize the system’s dynamic response. Once these amplitude components were obtained, the complete scattering wave amplitudes in the inlet, outlet, and expansion regions, as well as the vibrational modes of the membrane discs, were determined using the relations:


$$A_{n}=\frac{A_{n}^{s}+A_{n}^{a}}{2},\;C_{n}=\frac{B_{n}^{s}-B_{n}^{a}}{2},$$

$$B_{n}=\frac{B_{n}^{s}+B_{n}^{a}}{2},\;D_{n}=\frac{A_{n}^{s}-A_{n}^{a}}{2},$$

$$H_{1n}=\frac{H_{1n}^{s}+H_{1n}^{a}}{2},\;H_{2n}=\frac{H_{1n}^{s}-H_{1n}^{a}}{2}.$$

The incident, reflected, and transmitted acoustic energy components in the waveguide system can be computed in the inlet and outlet regions based on the definitions provided in [15]. The reflected energy at the inlet is obtained using reflection coefficients derived from the matching conditions, while the transmitted energy at the outlet is calculated from the transmission coefficients, assuming a normalized incident energy flow of unity. The corresponding expressions for the reflected and transmitted energy are:

$${ℰ}_{1}=\frac{a}{\alpha }\;Re\left[\sum_{n=0}^{N}|A_{n}{|}^{2}\eta_{n}X_{n}\right],$$ (64)

$${ℰ}_{2}=\frac{a}{\alpha }\;Re\left[\sum_{n=0}^{N}|D_{n}{|}^{2}\eta_{n}X_{n}\right].$$ (65)

It should be noted that energy conservation requires the total energy to satisfy ${ℰ}_{1}\;+\;{ℰ}_{2}={ℰ}_{3}=1$. Furthermore, the **transmission loss** due to the system can be evaluated using the ratio of transmitted to incident energy as:

$$\text{Transmission loss}=-10\log_{10}[{ℰ}_{2}],$$ (66)

as presented in [15]. Table 1 presents the convergence behavior of energy-related parameters for a cylindrical shell waveguide system characterized by specific material and geometric properties. The shell has a thickness of $\bar{h}=0.002$ m and is composed of a material with density $\rho_{s}=2700\;{\text{kg/m}}^{3}$, Young’s modulus $E=7.2\times 10^{10}\;{\text{N/m}}^{2}$, and Poisson’s ratio ν=0.34. The shell encloses a compressible fluid with density $\rho =1.2043\;{\text{kg/m}}^{3}$ and sound speed c=343m/s.

**Table 1:** Convergence of energies when $\bar{a}$ = 0.1 m, $\bar{b}$ = 0.2 m $\bar{L}$ = 0.05 m, *f* = 350 Hz.

*N*	${ℰ}_{1}$	${ℰ}_{2}$	${ℰ}_{3}$	*TL*
1	0.470293	0.529709	1	2.75962
6	0.470293	0.529709	1	2.75962
11	0.441876	0.558126	1	2.53267
16	0.442055	0.557947	1	2.53407
21	0.442039	0.557963	1	2.53394
31	0.442039	0.557963	1	2.53394
41	0.442039	0.557963	1	2.53394
51	0.442039	0.557963	1	2.53394
61	0.442039	0.557963	1	2.53394
71	0.442039	0.557963	1	2.53394

**Fig 2:**
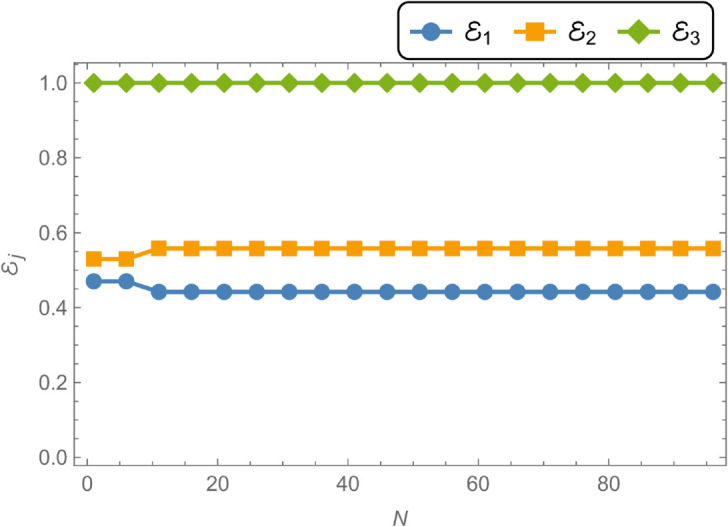
Energies against truncation parameter N.

**Fig 3:**
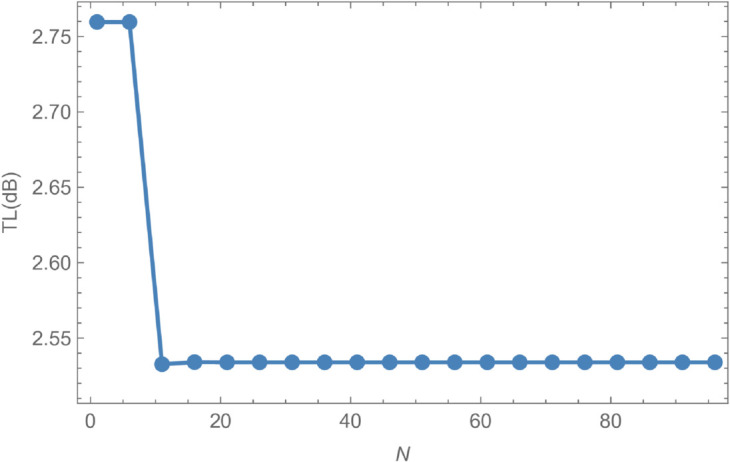
Transmission loss against truncation parameter N.

Geometrically, the shell has an inner radius of $\bar{a}=0.1$ m, an outer radius of $\bar{b}=0.2$ m, and a length of $\bar{L}=0.05$ m. The system is analyzed at an operating frequency of *f* = 350 Hz, with a membrane tension of *T* = 350 N. Numerical convergence is achieved by truncating the modal expansions to *N* = 50 terms, which ensures that the computed amplitude values are accurate to three decimal places. This underlines the importance of selecting an appropriate truncation parameter for reliable results. Fig 2 illustrates the evolution of transmitted and reflected amplitude components with increasing *N*, demonstrating convergence for both quantities when N≥21. The energy components shown in Fig 2 provide further insight: the reflected energy ${ℰ}_{1}$ stabilizes around 0.4 with minor oscillations, while the transmitted energy ${ℰ}_{2}$ remains steady near 0.6, indicating more efficient energy transmission compared to reflection. The total energy ${ℰ}_{3}={ℰ}_{1}+{ℰ}_{2}$ consistently equals unity across all values of *N*, thereby confirming energy conservation in the system. Fig 3 plots the transmission loss (TL), expressed in decibels (dB), as a function of the number of retained terms *N*. As *N* increases from 0 to approximately 20, the transmission loss decreases markedly from about 2.75 dB to 2.55 dB, reflecting improved energy transmission. Beyond *N* = 20, the transmission loss plateaus near 2.55 dB, exhibiting negligible variation with further increases in *N*. This behavior indicates that the system approaches a steady-state energy distribution, where additional modal terms yield diminishing returns. Overall, Fig 3 highlights the efficiency of wave transmission within the cylindrical shell and underscores the practicality of truncation in computational modeling.

To examine the pressure and velocity fields within a cylindrical chamber, we consider a configuration defined by the following geometric parameters: an inner radius $\bar{a}=0.1$ m, an outer radius $\bar{b}=0.2$ m, and a half-length $\bar{L}=0.05$ m. The analysis focuses on the behavior of these fields at the axial interfaces z=±L. The system is evaluated at an excitation frequency of *f* = 350 Hz and subjected to a membrane tension of *T* = 350 N. To accurately satisfy the clamped boundary conditions, maintain field continuity, and match membrane displacements, the series expansion was truncated at *N* = 100 terms.

**Fig 4:**
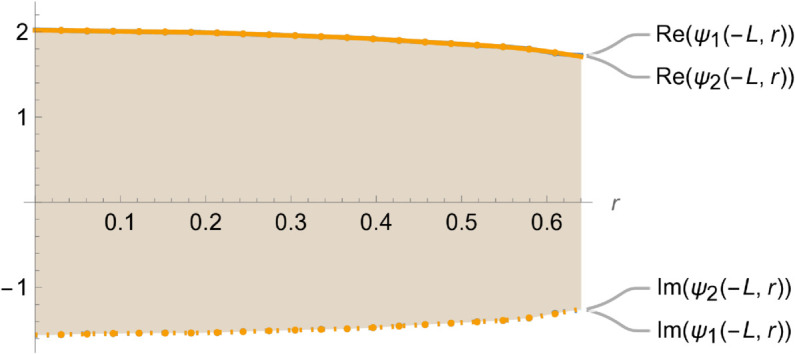
Real and imaginary parts of $\psi_{1}\left(r,z\right)$ and $\psi_{2}\left(r,z\right)$ at z=−L where $\bar{a}$ = 0.1m, $\bar{b}$ = 0.2m, $\bar{L}$ = 0.05, m f = 350Hz.

**Fig 5:**
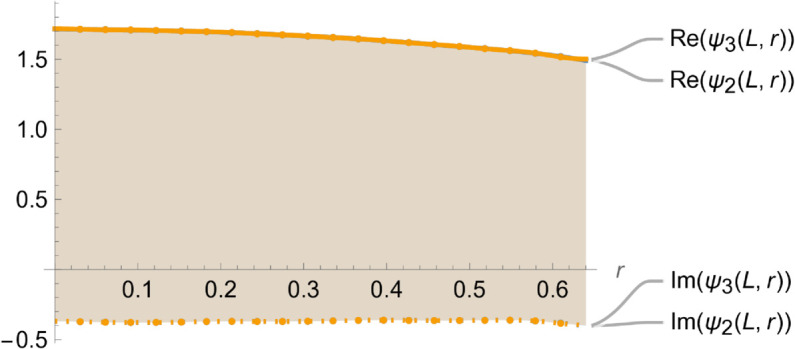
Real and imaginary parts of $\psi_{2}\left(r,z\right)$ and $\psi_{3}\left(r,z\right),$ at z = L where $\bar{a}$ = 0.1m, $\bar{b}$ = 0.2m, $\bar{L}$ = 0.05, m f = 350Hz.

**Fig 6:**
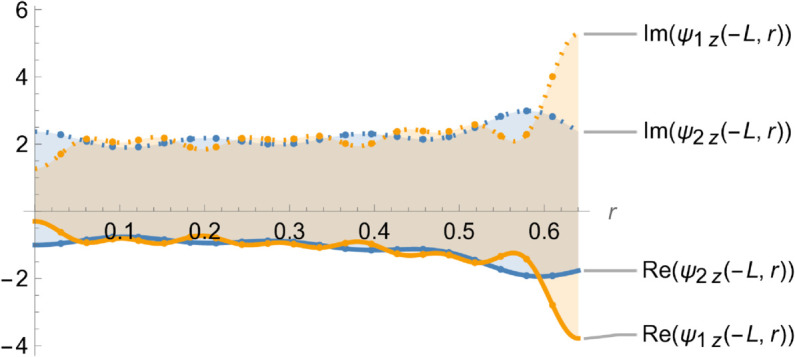
Real and imaginary parts of $\psi_{2z}\left(r,z\right)$ and $\psi_{1z}\left(r,z\right)$ at z = -L, where $\bar{a}$ = 0.1m, $\bar{b}$ = 0.2m, $\bar{L}$ = 0.05, m f = 350Hz.

**Fig 7:**
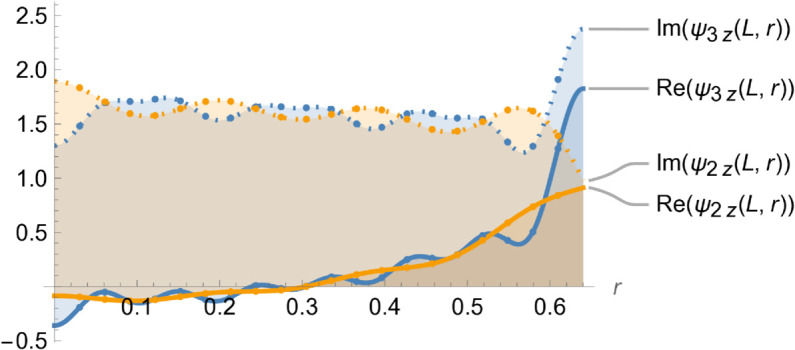
Real and imaginary parts of $\psi_{3z}\left(r,z\right)$ and $\psi_{2z}\left(r,z\right)$, at z = L where $\bar{a}$ = 0.1m, $\bar{b}$ = 0.2m, $\bar{L}$ = 0.05, m f = 350Hz.

Figs 4–7 illustrate the behavior of the matching conditions governed by Eqs (32)–(35), highlighting both the real and imaginary parts of the respective field components. Specifically, Fig 4 depicts the pressure continuity condition $\psi_{1}(r,z)=\psi_{2}(r,z)$ evaluated at z=−L,r=a, while Fig 5 shows the matching condition $\psi_{3}(r,z)=\psi_{2}(r,z)$ at z=L,r=a. Correspondingly, Figs 6 and 7 present the continuity of the axial velocity field, expressed as $\psi_{2z}(r,z)=\psi_{1z}(r,z)$ at z=−L,r=a and $\psi_{3z}(r,z)=\psi_{2z}(r,z)$ at z=L,r=a, respectively. The close alignment of the pressure and velocity fields at the interfaces affirms the continuity conditions imposed in the model, thereby validating the accuracy and consistency of the theoretical framework in capturing the physical behavior of the coupled wave–structure system.

**Fig 8:**
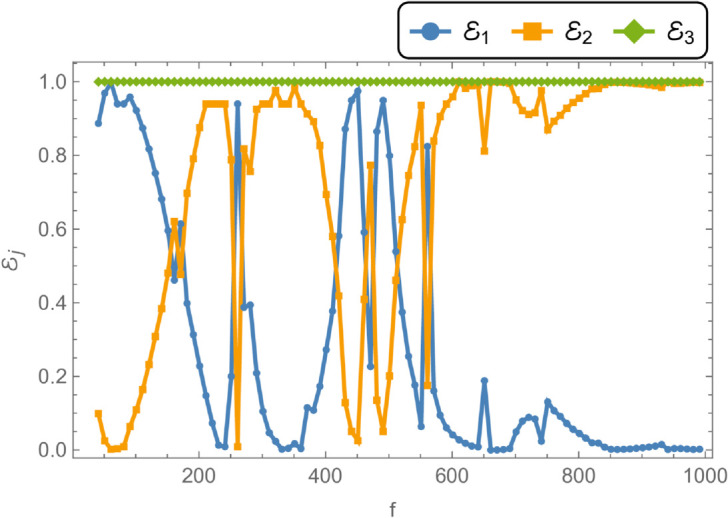
Energy components against frequency f, where, $\bar{a}$ = 0.3m, $\bar{b}$ = 0.5m, $\bar{L}$ = 0.1, m Tension = 350N.

**Fig 9:**
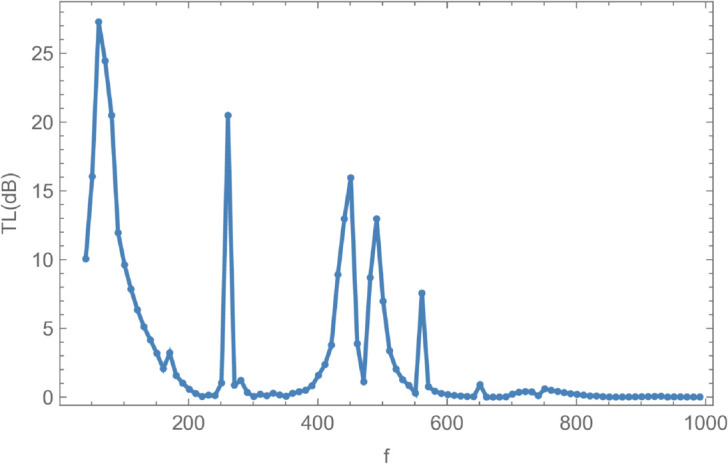
Transmission loss against frequency f, where, $\bar{a}$ = 0.3m, $\bar{b}$ = 0.5m, $\bar{L}$ = 0.1, m Tension = 350N.

To examine the influence of varying parameters on acoustic attenuation, simulations were conducted using a flexible cylindrical shell with an inner radius of $\bar{a}=0.3$ m, an outer radius of $\bar{b}=0.5$ m, and a half-chamber length of $\bar{L}=0.1$ m. The fluid potential amplitudes were calculated by truncating the modal expansion to *N* = 10 terms. Figs 8 and 9 present the variation of reflected, transmitted, and total energies over the frequency range 0≤f≤1000 Hz, under a membrane tension of *T* = 350 N. Notably, the total energy remains conserved at unity across the entire frequency range, indicating energy balance between the incident, reflected, and transmitted components.

**Fig 10:**
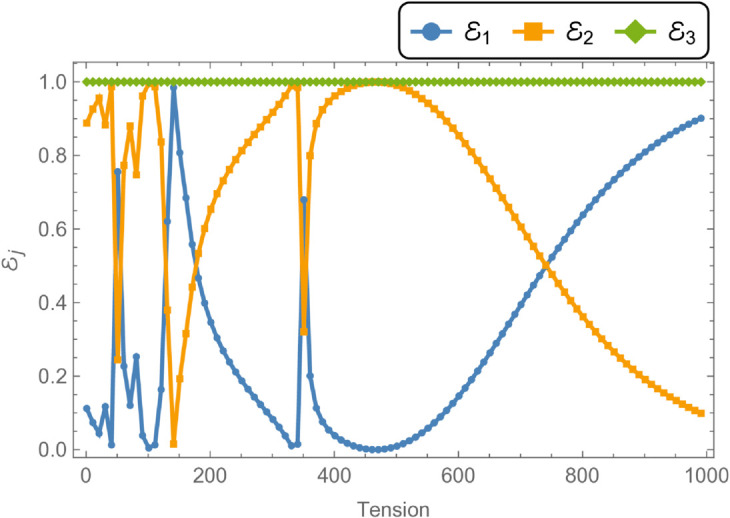
Energy components against tension T, where, $\bar{a}$ = 0.1m, $\bar{b}$ = 0.2m, $\bar{L}$ = 0.1, m f = 350N.

**Fig 11:**
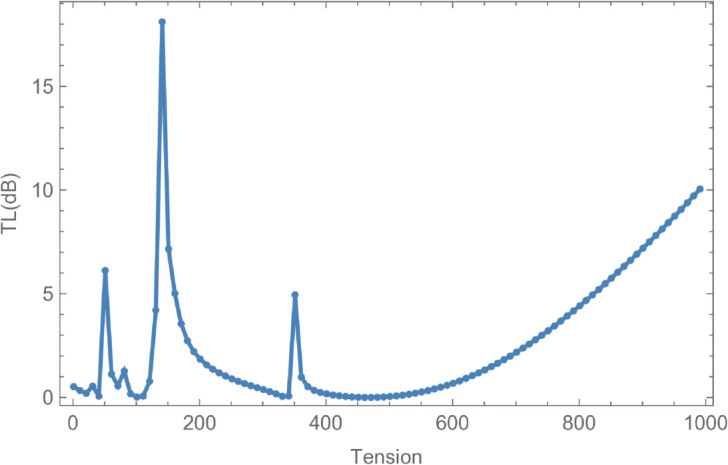
Transmission loss against tension T, where, $\bar{a}$ = 0.1m, $\bar{b}$ = 0.2m, $\bar{L}$ = 0.1, m f = 350N.

**Fig 12:**
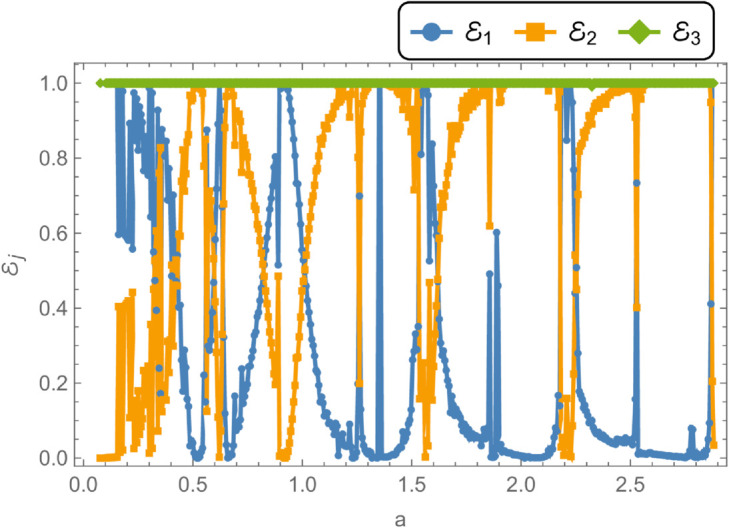
Energy components against radius a, where, $\bar{b}$ = 0.5m, $\bar{L}$ = 0.1, m f = 350Hz and tension T = 350N.

**Fig 13:**
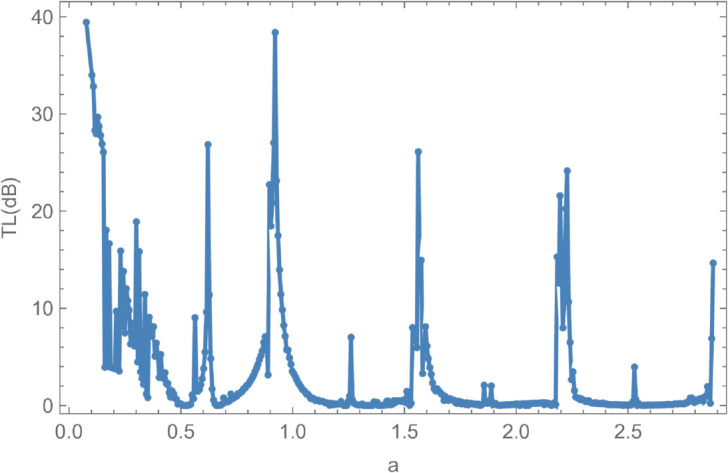
Transmission Loss against radius a, where, $\bar{b}$ = 0.5m, $\bar{L}$ = 0.1, m f = 350Hz tension T = 350N.

In Fig 8, the reflected energy displays a pronounced oscillatory behavior, characterized by multiple peaks and valleys, emphasizing its strong frequency dependence. The transmitted energy exhibits a similarly complex trend, with regions that appear to be inversely correlated with the reflected energy. For instance, at *f* = 50 Hz, a peak in reflected energy coincides with a dip in transmitted energy. At *f* = 170 Hz, both components attain equal magnitudes but with opposite phases, suggesting constructive or destructive interference. Cut-on modal behavior is observed around *f* = 240 Hz and *f* = 260 Hz. Additional increases in reflected energy are noted at *f* = 330 Hz, *f* = 370 Hz, *f* = 650 Hz, *f* = 700 Hz, and beyond *f*>850 Hz, accompanied by corresponding decreases in transmitted energy, thereby maintaining the total energy at unity. Fig 9 illustrates the transmission loss (TL) as a function of frequency. A sharp increase is observed near *f* = 50 Hz, followed by a reduction around *f* = 225 Hz, and a notable peak at *f* = 275 Hz. Beyond this point, transmission loss gradually declines with increasing frequency, reflecting a reduction in attenuation efficiency. To explore the influence of membrane tension on reflected and transmitted powers, their combined energy, and transmission loss, simulations were conducted for tension values ranging from 0≤T≤1000 N, as illustrated in Figs 10 and 11. The waveguide configuration consists of inlet, central, and outlet regions with radii $\bar{a}=0.1$ m and $\bar{b}=0.2$ m, and a half-length of $\bar{L}=0.1$ m. The frequency was fixed at *f* = 350 Hz, and the system was truncated to *N* = 10 terms for computational efficiency. Fig 10 reveals three prominent fluctuations in the scattering powers at approximately *T* = 140 N, *T* = 240 N, and *T* = 450 N. The amplitude of these oscillations increases with tension, and the spacing between the second and third fluctuations is roughly double that between the first and second. This progression leads to a transition from a narrowband to a broadband transmission loss profile, as observed in Fig 11. The broadening of the transmission loss peak is attributed to the increasing membrane tension, which suppresses membrane resonances and alters the energy scattering and transmission characteristics of the system. To examine the influence of duct dimensions on the scattering characteristics of a cylindrical waveguide, we analyzed the reflected and transmitted powers, along with the corresponding transmission loss, by varying the semi-inlet/outlet radius *r* = *a* from 0.01 m to 0.45 m. The analysis was performed under fixed conditions: outer radius $\bar{b}=0.5$ m, cavity half-length $\bar{L}=0.1$ m, frequency *f* = 350 Hz, and membrane tension *T* = 350 N. The results, presented in Figs 12 and 13, show that at small radii, a majority of the incident energy is reflected, as illustrated in Fig 12. As the radius *a* increases, the annular interface at z=±L expands, intensifying the geometric discontinuity. This increase in discontinuity leads to higher reflection levels, particularly near the cut-on modes of the expansion chamber. Notable fluctuations in reflected power occur at specific radii such as *a* = 0.6 m, 0.9 m, 1.6 m, and 2.2 m. In contrast, the transmitted power demonstrates an inverse trend relative to the reflected power. Fig 13 displays the corresponding transmission loss as a function of the radius *r* = *a*, which initially rises before declining, with values ranging between 0.1 dB and 0.6 dB. These variations are primarily attributed to the excitation of new cut-on modes within the expansion chamber, driven by energy leakage through the flexible shell structure bounded by elastic membranes at both ends.

**Fig 14:**
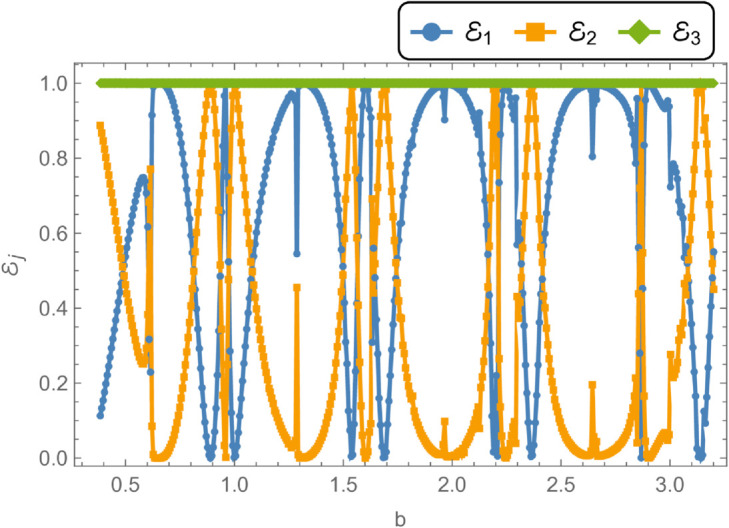
Energy components against radius b, where, $\bar{a}$ = 0.05m, $\bar{L}$ = 0.1, m f = 350Hz and tension T = 350N.

**Fig 15:**
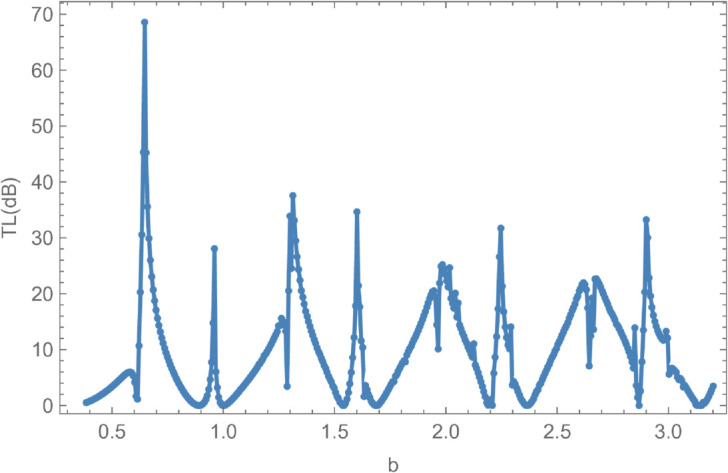
Transmission Loss against radius b, where, $\bar{a}$ = 0.05m, $\bar{L}$ = 0.1, m f = 350Hz and tension T = 350N.

Figs 14 and 15 present the reflected and transmitted powers, along with the transmission loss, as functions of the non-dimensional outer radius *b*, under fixed conditions: frequency *f* = 350 Hz, membrane tension *T* = 350 N, inner radius $\bar{a}=0.05$ m, and cavity half-length $\bar{L}=0.1$ m. The analysis investigates the behavior of these quantities as the cylindrical waveguide radius *r* = *b* varies from 0.06 m to 0.5 m. As *r* = *b* increases, the annular disc area at the interfaces z=±L expands, enhancing geometric discontinuities. Initially, the reflected power dominates over the transmitted power due to these discontinuities, with significant changes observed when new cut-on modes of the expansion chamber are excited. Fig 14 illustrates the interplay between reflected and transmitted energies, revealing an inverse relationship: increases in one correspond to decreases in the other, while their sum remains constant at unity, thereby confirming energy conservation. Fig 15 shows the transmission loss as a function of the radius *r* = *b*, highlighting a pronounced peak of approximately 67 dB at the onset of a new cut-on mode. These variations in transmission loss are primarily attributed to the excitation of additional cut-on modes in the expansion chamber, which arise due to energy leakage through the flexible shell structure bounded by elastic membranes.

**Fig 16:**
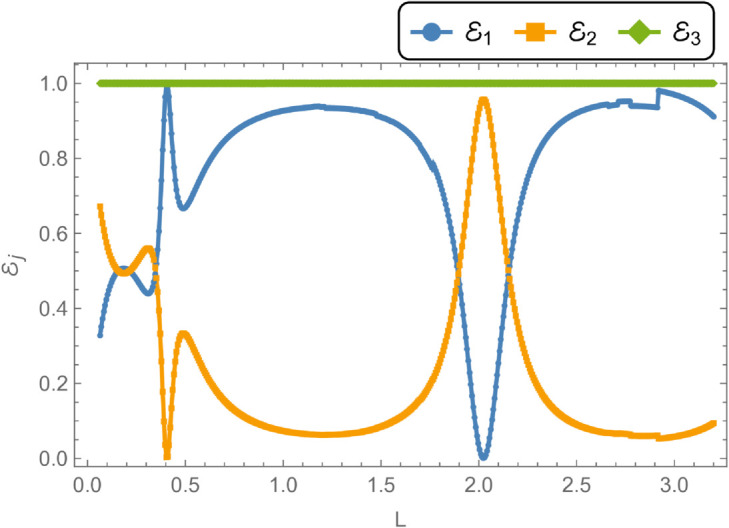
Energy components against length L, where, $\bar{a}$ = 0.1m, $\bar{b}$ = 0.2, m f = 350Hz and tension T = 350N.

**Fig 17:**
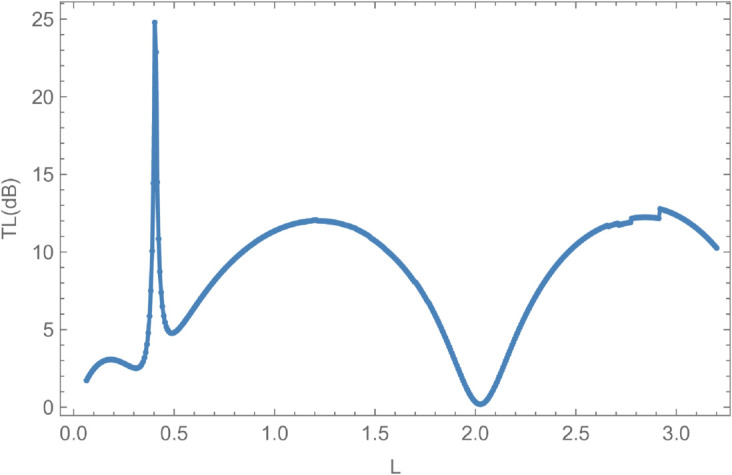
Transmission Loss against length L, where, $\bar{a}$ = 0.1m, $\bar{b}$ = 0.2, m f = 350Hz and tension T = 350N.

Figs 16 and 17 depict the variation of scattering powers and transmission loss as functions of the waveguide length *L*, for fixed parameters: inner radius $\bar{a}=0.1$ m, outer radius $\bar{b}=0.2$ m, frequency *f* = 350 Hz, and membrane tension *T* = 350 N. The analysis spans the range 0.01≤L≤0.5 m, focusing on reflected energy, transmitted energy, and transmission loss. In Fig 16, the reflected energy initially increases, reaching approximately 10% of its peak value, before stabilizing as *L* grows. The transmitted energy also exhibits notable fluctuations with varying *L*. A key feature occurs at *L* = 0.4 m, where reflected and transmitted energies intersect, with their sum equaling unity, marking the first cut-on point. Another cut-on is observed at *L* = 0.2 m, beyond which both energies increase before gradually declining. Fig 17 shows the corresponding transmission loss, which reaches a maximum of 25 dB at *L* = 0.4 m. As *L* continues to vary, the transmission loss declines, while reflected and transmitted energies evolve at differing rates. These behaviors underscore the sensitivity of acoustic energy distribution and attenuation to the axial length of the waveguide.

## 6 Summary and conclusion

This study examined acoustic wave propagation in a cylindrical shell waveguide system featuring a central expansion chamber and elastic membranes at both ends. The Mode Matching (MM) method was applied to derive expressions for symmetric and anti-symmetric modal amplitudes, while the Galerkin method was used to solve for membrane displacements based on the dynamic equations of motion. Continuity and boundary conditions were enforced at the membrane interfaces, and energy conservation principles were incorporated into the framework. Truncation of the infinite series to a finite number of terms (*N*) enabled efficient and accurate computation of the incident, reflected, and transmitted energy components.

Numerical results verified that the total energy—sum of reflected and transmitted components—remains constant across all examined scenarios, affirming both the accuracy of the truncated model and the validity of energy conservation. The convergence behavior supports the robustness of the solution in capturing the key acoustic dynamics of the system. The accuracy of the theoretical model was further validated through the close agreement of pressure and velocity fields at the interfaces. The consistent behavior of real and imaginary components demonstrated the model’s ability to satisfy continuity and boundary conditions reliably.

Parametric analyses revealed that variations in frequency, shell radius, membrane tension, and cavity length significantly influence wave scattering. Increasing shell radius enhanced geometric discontinuities at the membrane interfaces, resulting in elevated reflected energy and altered transmission characteristics, especially near cut-on modes. Similarly, increasing membrane tension suppressed membrane oscillations, broadened the bandwidth of transmission loss, and shifted the spacing between resonant scattering features. Transmission loss displayed strong dependence on frequency and structural parameters, with clear peaks near cut-on conditions. The reflected and transmitted energies often exhibited inverse trends, pointing to a coupled scattering mechanism influenced by the geometry and tension state of the membranes. Overall, the combined MM and Galerkin approach effectively captures the complex interaction of structural and acoustic fields in this waveguide system. The results provide actionable insights for optimizing design parameters—such as membrane tension and shell dimensions—to enhance energy transmission and control acoustic attenuation. These findings have direct applications in fields like noise reduction, duct acoustics, and ventilation system design.
